# Harlequin ichthyosis: A rare case

**DOI:** 10.4274/tjod.63004

**Published:** 2017-06-15

**Authors:** Belide Shruthi, B.R. Nilgar, Anita Dalal, Nehaben Limbani

**Affiliations:** 1 K.L.E. University’s Jawaharlal Nehru Medical College, Department of Obstetrics and Gynecology, Belagavi, India

**Keywords:** Harlequin ichthyosis, adenosine triphosphate binding cassette A12, autosomal recessive

## Abstract

Harlequin ichthyosis is a very rare condition that affects the skin of newborns. It is associated with poor barrier function of the skin leading to dehydration and leaves newborns prone to infections. It is due to mutations in adenosine triphosphate binding cassette A12 gene transmitted as an autosomal recessive disorder. The prognosis is very poor in these cases. Here, we report one such rare case.

## INTRODUCTION

Ichthyosis is derived from a Greek word, ichthys, which means fish. It refers to a fish-scale-like appearance of skin. Ichthyoses are disorders of skin characterized by dry, scaly and thickened skin. Ichthyosis may involve the skin alone or other organs also, and may be inherited or acquired. The mode of inheritance is autosomal or X-linked, and can be dominant or recessive. The 3 major types of autosomal recessive congenital ichthyosis are Harlequin ichthyosis, lamellar ichthyosis, and congenital ichthyosiform erythroderma. Harlequin ichthyosis is the least common and most severe form.

The first case was reported in South Carolina, United States of America, in 1750 by Hart^(1)^. The first case diagnosed antenatally was reported in 1983^(2)^. Antenatal diagnosis in suspected cases can be confirmed using electron microscopy of fetal skin biopsy and DNA-based diagnosis with chorionic villus sampling or amniocentesis^(3)^. There is no cure for this condition and only supportive treatment can be given to prolong life.

## CASE REPORT

A primigravida woman aged 20 years registered with a private practitioner, reported to the labor room with 33 weeks’ of gestation with preterm premature rupture of membranes in latent labor with breech presentation. A history of 2^nd^ degree consanguinity was noted with 9 months of married life. An earlier scan detected polyhydramnios. The other abnormalities were not appreciated on the scan. She underwent emergency cesarean section in view of footling presentation and a female baby weighing 1.9 kg was delivered on December 22^nd^, 2016.

**Figure 1:**
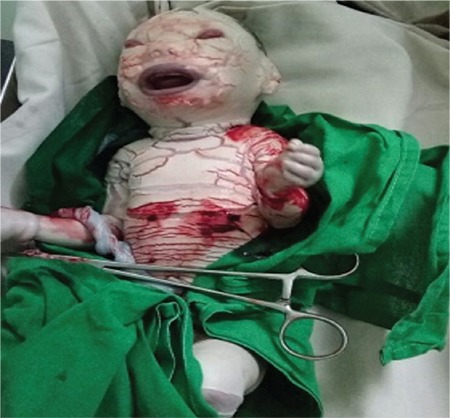
Skin of the baby at birth showing armor-like covering with creases

**Figure 2:**
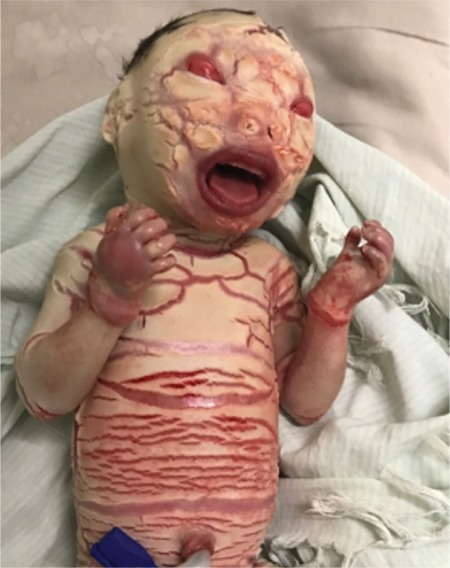
Bleeding from the creases with ectropion and thick lips with mouth open

**Figure 3:**
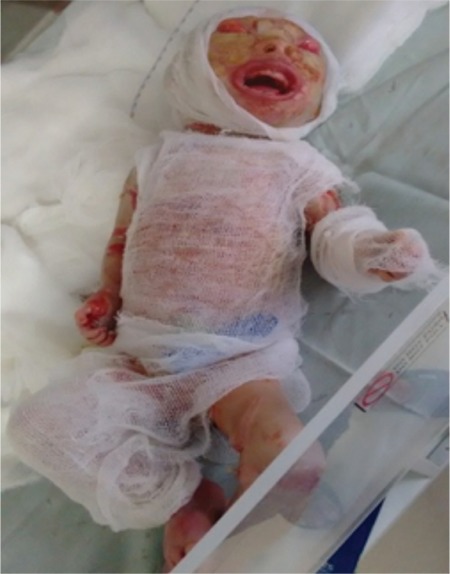
Ectropion, open mouth and thick lips seen. Emollients applied and the baby covered with sterile gauze

**Figure 4:**
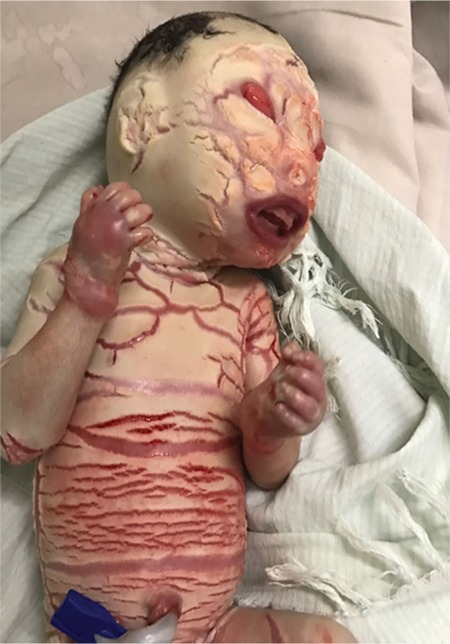
Ears are small with closed pinna. Fingers showing flexion deformity

The baby had white porcelain-like skin covering the body like armor with deep creases all over the body as shown in Figure 1. Bleeding was noticed from the creases. The baby had a weak cry at birth. Eyelids and lips were everted showing ectropion and eclabion, respectively. Nasal hypoplasia with two nostrils was seen. The mouth was open with thick lips as seen in Figures 2 and 3. The ears were small with closed pinna. The fingers and toes were flexed and fixed flexion deformity noticed, as seen in Figure 4. The heart rate and respiratory rate were normal. The baby was sent to the neonatal intensive care unit for further management.

Later, the baby’s skin was noticed to peel off leaving erythematous fissures. A peripheral intravenous line could not be secured and the umbilical vein was accessed. Conservative management was given with intravenous antibiotics, emollients, and retinoids. Feeds were given through a Ryles tube. The baby died on the 3^rd^ day. The parents refused an autopsy and skin biopsy of the baby.

The parents were called for genetic counseling.

## DISCUSSION

Harlequin ichthyosis is also known as Harlequin baby syndrome/Harlequin fetus syndrome/ichthyosis congenita. The word Harlequin is derived from a similar appearance of a comic servant character. Harlequin ichthyosis is a rare form of congenital ichthyosis with an overall incidence of 1 in 300,000 births^(3)^. Approximately 200 cases have been reported throughout the world^(4)^. Recently, one case was reported in June 2016 at Nagpur, India, published in the International Journal of Pharmacy^(5)^. It is inherited as an autosomal recessive condition^(3)^. It can affect male and female children equally. It occurs due to a mutation in the adenosine triphosphate binding cassette A12 (ABCA12) gene, which is responsible for the exocytosis of lipid-containing lamellar granules, which control the process of desquamation^(6)^. The locus for the ABCA12 gene lies on chromosome 2q35^(7)^. The major types of mutations responsible for this are nonsense mutations or frameshift mutations^(7)^. This condition can be diagnosed antenatally by scanning with the following features: polyhydramnios (seen in this case), echogenic amniotic fluid, fetal growth restriction, eyes closed with eversion of the eyelids and lips (ectropion and eclabion, respectively), flat nose, mouth wide open, ears not well formed, flexion of extremities, mottled, breeched skin of the face and limbs, hyperflexion of fingers and toes, absence of opening movements of fingers^(1)^. A 3D scan is better for diagnosing this condition.

Life-threatening complications include dehydration, supervening infections, and respiratory insufficiency. The prognosis is very poor. Most affected babies do not survive beyond the first week of life. It has been reported that the survival rate varies from 10 months to 25 years with supportive treatment depending on the severity of the condition^(8)^. Recurrence of this condition in the next pregnancy is 25%^(1)^. Genetic counseling should be undertaken for these cases.

## References

[1] Nayak S, Dash SP, Khatua M (2015). Fetal Harlequin Ichthyosis - A Case Report. Journal of Dental and Medical Sciences.

[2] Bongain A, Benoit B, Ejnes L, Lambert JC, Gillet JY (2002). Harlequin fetus: three-dimensional sonographic findings and new diagnostic approach. Ultrasound Obstet Gynecol.

[3] Jilumudi UB (2012). Harlequin Ichthyosis: A medico legal case report & review of literature with peculiar findings in autopsy. J Forensic Leg Med.

[4] Baby C (2016). Harlequin Ichthyosis: A disease chronic rather than fatal. World Journal of Pharmacy and Pharmaceutical Sciences.

[5] Pitchaiah G (2016). Harlequin baby syndrome- a rare autosomal recessive skin disorder. IJRPC.

[6] Hovnanian A (2005). Harlequin Ichthyosis unmasked: a defect of lipid transport. J Clin Invest.

[7] Thomas AC, Cullup T, Norgett EE, Hill T, Barton S, Dale BA, et al (2006). ABCA12 Is the Major Harlequin Ichthyosis Gene. J Invest Dermatol.

[8] Rajpopat S, Moss C, Mellerio J, Vahlquist A, Ganemo A, Hellstrom-Pigg M, et al (2011). Harlequin ichthyosis: a review of clinical and molecular findings in 45 cases. Arch Dermatol.

